# Evaluation of Smartphone Applications for Cardiopulmonary Resuscitation Training in South Korea

**DOI:** 10.1155/2016/6418710

**Published:** 2016-09-07

**Authors:** Chiwon Ahn, Yongtak Cho, Jaehoon Oh, Yeongtak Song, Tae Ho Lim, Hyunggoo Kang, Juncheol Lee

**Affiliations:** ^1^Department of Emergency Medicine, College of Medicine, Hanyang University, Seoul, Republic of Korea; ^2^Convergence Technology Center for Disaster Preparedness, Hanyang University, Seoul, Republic of Korea

## Abstract

*Objective*. There are many smartphone-based applications (apps) for cardiopulmonary resuscitation (CPR) training. We investigated the conformity and the learnability/usability of these apps for CPR training and real-life supports.* Methods*. We conducted a mixed-method, sequential explanatory study to assess CPR training apps downloaded on two apps stores in South Korea. Apps were collected with inclusion criteria as follows, Korean-language instruction, training features, and emergency supports for real-life incidents, and analyzed with two tests; 15 medical experts evaluated the apps' contents according to current Basic Life Support guidelines in conformity test, and 15 nonmedical individuals examined the apps using System Usability Scale (SUS) in the learnability/usability test.* Results*. Out of 79 selected apps, five apps were included and analyzed. For conformity (ICC, 0.95, *p* < 0.001), means of all apps were greater than 12 of 20 points, indicating that they were well designed according to current guidelines. Three of the five apps yielded acceptable level (greater than 68 of 100 points) for learnability/usability.* Conclusion*. All the included apps followed current BLS guidelines and a majority offered acceptable learnability/usability for layperson. Current and developmental smartphone-based CPR training apps should include accurate CPR information and be easy to use for laypersons that are potential rescuers in real-life incidents.* For Clinical Trials*. This is a clinical trial, registered at the Clinical Research Information Service (CRIS, cris.nih.go.kr), number KCT0001840.

## 1. Introduction

Sudden cardiac arrest (SCA) remains a leading cause of death in developed countries, including South Korea, despite efforts devoted to prevention of SCA [1–3]. Although there are many factors that dictate the outcomes of SCA, it is well known that survival rates are up to three times higher when cardiopulmonary resuscitation (CPR) is performed immediately after SCA [4, 5]. Various methods for demonstrating high quality CPR and immediate recognition of cardiac arrest, including face-to-face training and video-based instruction for bystanders, have yielded improvement in participation rates during incidents of SCA [6, 7]. However, only 12–42% of cardiac arrest patients witnessed by the layperson received CPR during out-of-hospital cardiac arrest (OHCA) [8–10]. The low rate of layperson intervention may be due to a failure to recognize cardiac arrest or a lack of confidence due to insufficient CPR training/education [11].

Recently, many medical and healthcare applications (apps) have been developed and registered in online mobile apps stores, because there is no limitation in time and space [12, 13]. In particular, a number of smartphone-based apps have been developed by public institutions and companies in order to enhance CPR education [14–17]. Smartphone-based apps could be an important and epochal medium, as they overcome the limitations of traditional CPR training and remind users, particularly layperson, of CPR. However, one flaw in app-based CPR training and education is that some apps may not adequately reflect current guidelines, potentially resulting in the transmission of incorrect information. Even some apps adhering to current guidelines may not be useful for layperson, as the apps might be difficult to operate and users may have low interest in their use. In one study, Kalz et al. [14] reported that very few apps reflect current BLS guidelines and offer an acceptable level of usability for layperson rescue.

As of January 2016, 85.2% of the South Korean population owned smartphones, a number that is steadily on the rise [18]. Additionally, many smartphone-based CPR training apps have been downloaded in South Korea, though no study has systematically investigated the CPR training apps. We assessed the conformity of smartphone-based CPR training apps to current CPR guidelines and evaluated the learnability and usability of the apps in incidents of SCA.

## 2. Materials and Methods

### 2.1. Setting and Participants

This mixed-method, sequential explanatory design study was approved by the Institutional Review Board of Hanyang University Hospital (Seoul, South Korea) (IRB HYUH2015-08-012-001) and was conducted in September 2015. The mixed-methods sequential design consisted of identification of smartphone-based CPR training apps, examination of conformity of apps to the 2010 American Heart Association Basic Life Support (AHA BLS) guidelines, and learnability and usability testing. Fifteen AHA BLS-certified healthcare providers and fifteen laypersons with no CPR training were recruited for the first and second phases of the study, respectively. Participants were recruited voluntarily by a notice on a bulletin from September 21, 2015, to September 30, 2015. Each potential participant received written information regarding the purpose of the study, and all participants provided written informed consent.

### 2.2. Materials and Experimental Methods

A Galaxy S4 smartphone (Samsung Electronics Co., Seoul, South Korea) with android (mobile operating system of Google) and an iPhone 5 (Apple Inc., Cupertino, CA, USA) with iOS (mobile operating system of Apple) were used for our investigation. Both mobile operating systems have a 99.8% market share in Korea (iOS 23.1% and android 76.7%) [19]. Therefore, we searched for and identified mobile apps from the Google Play Store and the Apple App Store, the two largest online stores for mobile apps (as of September 2015). Search terms included were “cardiopulmonary resuscitation” OR “CPR” OR “chest compression” OR “basic life support” in both English and Korean languages. In South Korea, the proportion of trueborn Korean is 97.8% [20], and almost all use and speak Korean language with low diversity of languages [21]. Therefore, we excluded apps with no Korean language in screening. And selected versions in the Google Play Store that were also present in the Apple App Store. From the selected apps, we excluded apps that did not contain CPR-related content and had error for operation of apps. Finally, we included apps that contained the following features: (1) training features and (2) emergency support for real-life incidents. “Emergency support for real-life incidents” means that layperson could be served guidance or accurate information for CPR within apps in real cardiac arrest situation; we selected this as mandatory feature. The identification and selection of apps included in this study are based on Preferred Reporting Items for Systematic Reviews and Meta-Analysis (PRISMA) guidelines [22].

*First Test: Conformity Test of Smartphone-Based CPR Apps to 2010 AHA BLS Guidelines Checklist*. We made the* conformity *checklist using the AHA BLS checklist for education by authors. The* conformity* checklist contained 10 items as follows: (1) how to check a patient's response and abnormal breathing, (2) how to activate the emergency medical system, (3) how to get someone to bring an automatic external defibrillator (AED), (4) correct CPR sequence (chest compression, airway, breathing, C-A-B), (5) existence of hands-only CPR for lay-rescue, (6) how to begin CPR rapidly, (7) proper compression position of the chest (i.e., lower half of the sternum), (8) adequate chest compression depth (i.e., at least 5 cm or 5-6 cm), (9) proper chest compression rate (i.e., at least 100 or 100–120 numbers/min), and (10) mention of complete chest decompression. Each item was scored on a numeric scale (0; nonexistent or incorrect information, 1; insufficient information, 2; sufficient information), with a maximum possible score of 20 points. 

*Second Test: Learnability and Usability Test of Smartphone-Based CPR Training Apps Using the System Usability Scale (SUS)*. For learnability and usability evaluations, we used the modified System Usability Scale (SUS), a simple but reliable method for evaluating the usability of a technological product or service [23–25]. The SUS consists of 10 questions: five positively worded questions (odd-numbered domain) and five negatively worded question (even-numbered domain) as follows:

*Modified System Usability Scale (SUS) Questions*

1. I think that I would like to use this product frequently.
2. I found the product unnecessarily complex.
3. I thought the product was easy to use.
4. I think that I would need the support of a technical person to able to use this product.
5. I found the various functions in this product were well integrated.
6. I thought there was too much inconsistency in this product.
7. I would imagine that most people would learn to use this product very quickly.
8. I found the system very awkward to use.
9. I felt very confident using the product.
10. I needed to learn a lot of things before I could get going with the product.

Questions (4) and (10) represent a value of learnability for laypersons, while the other questions represent a value of usability. The SUS showed the domains as five scales numbered from 1 (strongly disagree) to 5 (strongly agree). To obtain a score, the following formulas are used:

1. Positively worded domains = (score – 1).
2. Negatively worded domains = (5 – score).
3. After summing then ten domains, multiply by 2.5 =* total SUS*.

### 2.3. Data Collection

We recorded background information pertinent to the apps included in this investigation. Basic information consisted of (1) manufacturer, (2) number of downloads, (3) purchase cost, (4) last update, (5) type of content (video instruction, text instruction, audio instruction, video simulation, animation, and graphics), (6) purpose of the app, (7) underlying guideline, (8) target user (including pediatric), (9) detection of AED location, (10) supply of auditory guidance, (11) feedback system (compression rate and/or depth), and (12) direct connection to activate for Emergency Medical Service (EMS).

In the conformity test, each participant had 10 minutes of evaluation time for each app and five minutes of resting time before each evaluation. In the SUS learnability and usability test, each layperson had 30 minutes of evaluation time for each app and 10 minutes of resting time before each evaluation in the silent room with one observer. If layperson was not familiar with the device or had problems operating or controlling the device, observer helped them providing guidance. The order in which apps were evaluated was randomized for each participant.

### 2.4. Statistical Analysis

Data were compiled using a standard spreadsheet program (Excel; Microsoft, Redmond, WA, USA) and were analyzed using the Statistical Package for the Social Sciences (SPSS) 18.0 for Windows (SPSS Inc., Chicago, IL, USA). We generated descriptive statistics, and data are presented as the mean ± standard deviation (SD). We calculated an intraclass correlation coefficient (ICC) for all questions in tests of both phases. *p* values of < 0.05 were considered statistically significant. In the conformity test, we assessed the results by 5 Likert scales: (1) very high; 20 ≥ score ≥ 16 points, (2) high; 16 > score ≥ 12, (3) moderate; 12 > score ≥ 8, (4) low; 8 > score ≥ 4, (5) very low; 4 > score ≥ 0. A mean score of SUS > 68 is an acceptable value of learnability and usability, based on the current literature [14, 26].

## 3. Results

### 3.1. Apps Selection

**Figure 1:**
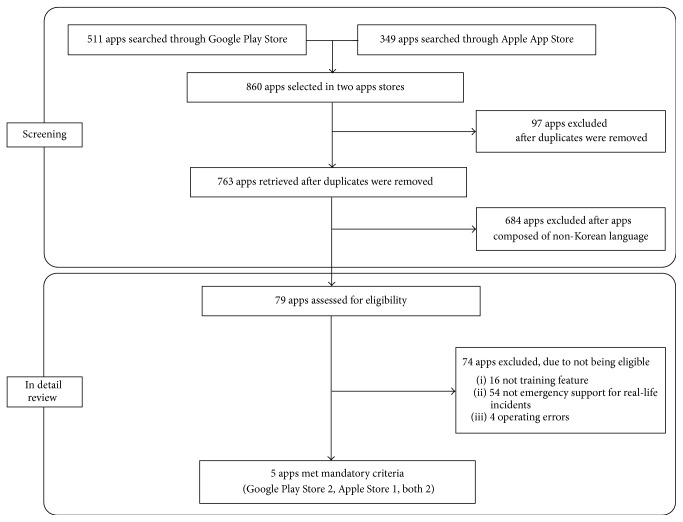
Flowchart of apps screening and selection.

A total of 511 apps and 349 apps were identified through the Google Play Store and the Apple App Store, respectively. After removing duplicates, we selected apps that consisted of CPR training in the Korean language. 79 apps were retrieved after screening, and then we operated and evaluated these apps in detail. 16 apps did not have CPR training feature, 54 apps did not have emergency support for real-life incidents, and 4 apps had errors when they were operated. Finally, five apps met our mandatory criteria (Figure 1). Two apps were registered in the Google Play Store, one was registered in the Apple App Store, and two were registered in both stores. Notable attributes of these apps are presented in Table 1, including basic app information, mandatory features, and feedback systems. Three apps had auditory guidance for the compression rate (by metronome), and only the “UCPR” app had feedback systems for the compression rate and depth (by accelerometer). There were no apps for pediatric BLS.

### 3.2. Results of Conformity to CPR Guidelines

**Figure 2:**
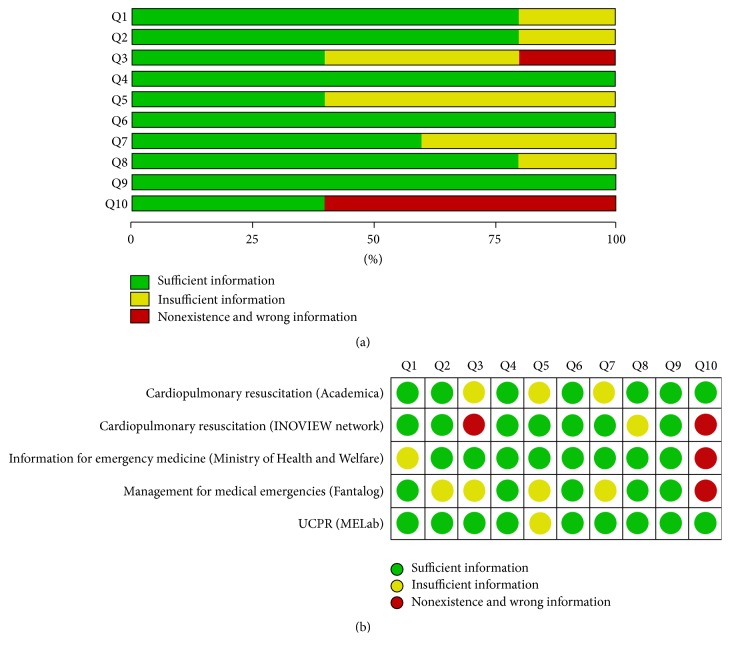
Analysis of information fulfillment for the* conformity* checklist. Fulfillment of sufficient information (a) in each question and (b) in each app. Q1, how to check the patient's response and abnormal breathing; Q2, how to activate the emergency medical system; Q3, how to get someone to bring an automatic external defibrillator (AED); Q4, correct cardiopulmonary resuscitation (CPR) sequence [chest compression, airway, breathing, C-A-B]; Q5, existence of hands-only CPR for lay-rescue; Q6, how to begin the CPR rapidly; Q7, proper compression position of chest (i.e., lower half of sternum); Q8, adequate chest compression depth (i.e., at least 5 cm or 5-6 cm); Q9, proper chest compression rate (i.e., at least 100 or 100–120 numbers/minute); Q10, mention of complete chest decompression.

The intraclass correlation (ICC) for the* conformity* checklist was 0.95 (*p* < 0.001, 95% Confidence Interval (CI) 0.93–0.97). The results of conformity to AHA 2010 BLS guideline testing are shown in Table 2. The apps we investigated, whose mean scores in conformity to the AHA 2010 BLS guidelines evaluation (in parentheses) were as follows: “UCPR” (MELab) (17.80 ± 1.01), “cardiopulmonary resuscitation” (Academica) (16.40 ± 1.88), “information for emergency medicine” (Ministry of Health and Welfare) (16.13 ± 1.24), “cardiopulmonary resuscitation” (INOVIEW network) (14.73 ± 1.09), and “management for medical emergencies” (Fantalog) (13.47 ± 2.94). Analyses of the conformity scores for each question are shown in Figure 2. Three questions fulfilled all of the apps (Q4, Q6, and Q9). Three apps did not fulfill “mention of complete chest decompression” (Q10).

### 3.3. Results of Learnability and Usability Evaluation Using SUS

**Figure 3:**
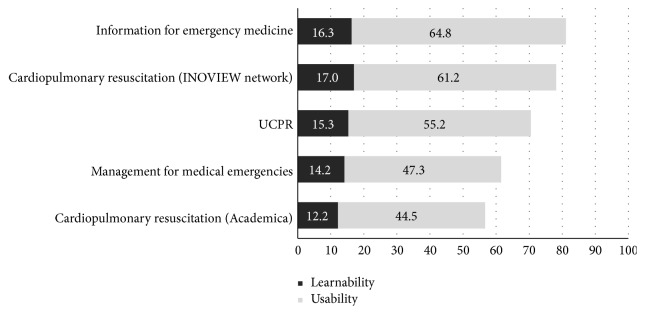
Mean learnability and usability testing scores of five apps using the System Usability Scale (SUS).

Three apps earned well over 68 points in learnability and usability testing, and the “information for emergency medicine” app had the highest score (81.17 points) (Table 2). For learnability, the “cardiopulmonary resuscitation” (INOVIEW network) app had 17.00 points, less than one point more than the “information for emergency medicine” app (16.30 points) (Figure 3).

## 4. Discussion

Although there are various methods for CPR training, most methods are not comprehensive [27]. Effectiveness and accuracy of CPR training are important factors, and retention of skills and knowledge is essential [28]. A reminder CPR video clip on a mobile phone was effective for education retention by trainees at three months after initial training [29]. A CPR animation instruction on a mobile phone was also effective in checklist assessment and time-interval compliance in trainees [30]. Smartphones are easy to access for civilians, and smartphone-based apps could provide both text and video clips for CPR. Alternatively, CPR training apps could be used for both CPR training and education retention after training. In this study, five of 79 smartphone-based CPR training apps met our mandatory criteria. The total download numbers of CPR training apps have been counted to be about several hundred thousands, ranging from about 1,000 to 1,000,000 times for each app. CPR training apps with incorrect or insufficient CPR information could result in layperson unintentionally harming the victim in real incidents. Thus, these apps should be examined by experts prior to public release.

Smartphone-based CPR training apps could provide auditory guidance through speakers, feedback for high quality chest compression using accelerometers, and the nearest AED location using global positioning system (GPS) sensors [15–17, 31]. Three of the five apps we examined incorporated auditory guidance of chest compression rate using a metronome, though just one app had an audiovisual feedback system for both the chest compression rate and depth (using an accelerometer). Several simulation studies have demonstrated that both smartphones and smartwatches with an accelerometer could be good alternative devices [32–34]. Two of the five apps were able to locate the nearest AED. The addition of audiovisual feedback is advisable in smartphone-based CPR training apps.

We included support for pediatric BLS as a special feature of this study. However, no app solely supports pediatric BLS. Although there are few pediatric arrest patients compared to adults, CPR training apps should also include an explanation of pediatric BLS.

All five apps analyzed in this study were designed well, yielding more than 12 points in conformity testing. For the tenth question, however, only two apps had sufficient explanation of complete chest decompression, which is a factor as important as chest compression [35, 36]. In October 2015, international CPR guidelines were changed, and CPR training apps should be updated according to new guidelines [36, 37]. High scores on the SUS scale indicate that the product or service is easy for the user to learn and handle. Three of the five apps we examined yielded SUS scores greater than 68 points. Some apps with high scores in conformity testing did not yield high scores in learnability and usability testing. In the future, easy-to-use, accurate CPR training apps should be developed.

There are several limitations to this study. First, user interest in CPR training apps improves educational transmission, and we did not attempt to find the interest factor in this study [14]. Second, the resident population of foreigners in Korea is growing every year, according to the South Korean Census. An investigation of CPR training apps that consist of various languages would be required for further examination. Not all CPR training apps evaluated in this study offered training in other languages. Finally, we conducted this study with two types of smartphones. An individual's skill or familiarity with a particular type of smartphone might have biased learnability and usability scores.

## 5. Conclusion

In conclusion, five CPR training apps followed current BLS guidelines, and three offered an acceptable level of learnability and usability for layperson. Current and developmental smartphone-based CPR training apps should include accurate CPR information (considering new international guidelines) and should be easy to use for laypeople that are potential rescuers in real-life incidents of SCA.

## Figures and Tables

**Table 1:** Characteristics of included apps.

Title	Cardiopulmonary resuscitation (CPR)	Cardiopulmonary resuscitation	Information for emergency medicine	Management for medical emergencies	UCPR
App information					
Manufacturer	Academica	INOVIEW network	Ministry of Health and Welfare	Fantalog Interactive Co., Ltd.	MELab
Icon image	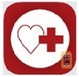	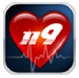	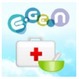	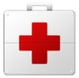	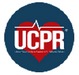
Number of downloads	Unknown	10,000–50,000 (android)	500,000–1,000,000 (android)	500,000–1,000,000	1,000–5,000
Purchase cost	Free	Free	Free	Free	Free
Last update	March 5, 2013	April 24, 2013	July 7, 2015	June 8, 2011	December 3, 2014
Distributor	iOS	Android, iOS	Android, iOS	Android	Android
Language	Korean	Korean	Korean	Korean	Korean
Mandatory features					
Training feature					
Educational video or animations	—	O	O	—	O
Real-incident animation instructions	—	O	—	—	—
Real-incident picture instructions	O	O	O	O	O
Real-incident audio instructions	—	O	O	—	O
Emergency support for real incidents	O	O	O	O	O
Special features					
AED location	—	—	O	—	O
Auditory guidance	—	O	O	—	O
Feedback					
Compression rate	—	—	—	—	O
Compression depth	—	—	—	—	O
Pediatric BLS	—	—	—	—	—
Direct connection to activate for Emergency Medical Service (EMS)	O	O	O	O	O

**Table 2:** Mean, standard deviation, and rank of *conformity* checklist score to the AHA 2010 BLS guidelines and modified System Usability Scale (SUS) score.

Title (manufacturer)	Conformity		Learnability and usability	
	Mean ± SD	Rank	Mean, SD	Rank
Cardiopulmonary resuscitation (Academica)	16.40 ± 1.88	2	56.67 ± 23.58	5
Cardiopulmonary resuscitation (INOVIEW network)	14.73 ± 1.09	5	78.17 ± 20.49	2
Information for emergency medicine (Ministry of Health and Welfare)	16.13 ± 1.24	3	81.17 ± 19.01	1
Management for medical emergencies (Fantalog)	13.47 ± 2.94	4	61.50 ± 19.54	4
UCPR (MELab)	17.80 ± 1.00	1	70.50 ± 24.33	3

SD: standard deviation.
